# Transferring information without distortion

**DOI:** 10.7554/eLife.41894

**Published:** 2018-10-25

**Authors:** Steven S Andrews, Roger Brent, Gábor Balázsi

**Affiliations:** 1 Division of Basic SciencesFred Hutchinson Cancer Research CenterSeattleUnited States; 2 Department of PhysicsSeattle UniversitySeattleUnited States; 3 Louis and Beatrice Laufer Center for Physical & Quantitative BiologyStony Brook UniversityStony BrookUnited States; 4 Department of Biomedical EngineeringStony Brook UniversityStony BrookUnited States

**Keywords:** signaling pathways, linearity, signal transmission, human cell lines, signaling systems, Other

## Abstract

Despite employing diverse molecular mechanisms, many different cell signaling systems avoid losing information by transmitting it in a linear manner.

**Related research article** Nunns H, Goentoro L. 2018. Signaling pathways as linear transmitters. *eLife* **7**:e33617. doi: 10.7554/eLife.33617

Much as explorers might use compasses and altimeters to establish where they are and radios to communicate with one another, the cells building a multicellular organism sense chemical gradients to determine their position and send and receive chemical signals to communicate. This sensing and communication works best if signals get transmitted into the cell’s interior without any loss of information (Figure 1A). In the early 20th century, the molecular explanation for such signaling seemed simple: Hill and others found that the physiological response to a drug increased with dose in a manner that could be explained by the kinetic theory of ligand-receptor binding. This and other reasoning led to the idea that drugs (ligands) bind to specific protein molecules called receptors, which directly produce the cellular response (Clark, 1933). In this picture, signaling was necessarily linear and hence undistorted: for example, doubling the proportion of ligand-bound receptors would double the cellular response.

**Figure 1.**
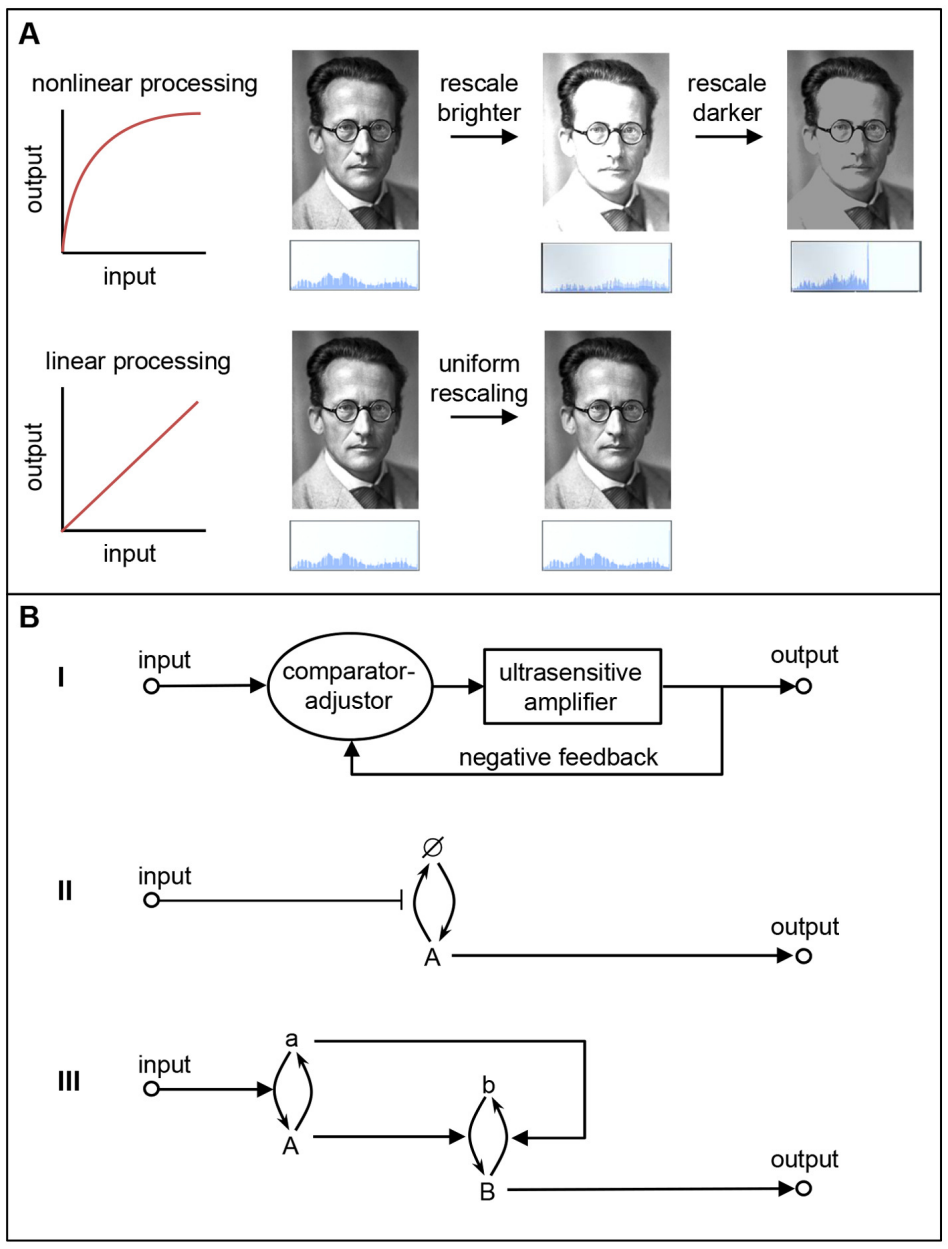
Linear and nonlinear signal processing. (**A**) Linear signal processing preserves information, whereas nonlinear signal processing does not. This can be seen by rescaling the gray-scale values in this image of Schrödinger with nonlinear signaling (top) and linear signaling (bottom). The nonlinear approach leads to saturation at high input levels, which means that some information is irretrievably lost, so the original image cannot be retrieved by reversing the rescaling process. The linear approach avoids these problems because the outputs are equal to the inputs, so information is not lost (Yu et al., 2008). The histograms below the images show the distribution of brightness values in the images. (**B**) The three linear signaling mechanisms that we are aware of. I) A mechanism that combines an ultrasensitive amplifier with negative feedback; this is used by EGF-ERK. II) A mechanism that modulates an unsaturated or unsaturable cycle: in this case the cycle involves protein synthesis (to form A), degradation (to produce Ø), resynthesis (to form A again), and so on: this mechanism is apparently used by the canonical Wnt and TGF-β pathways. III) A push-pull mechanism in which the active form of one protein (A) stimulates downstream activity (b → B) while the nominally inactive form (a) acts to reduce downstream activity (B → b). The signaling pathways examined by Nunns and Goentoro – the canonical Wnt, EGF-ERK and TGF-β pathways – appear to use the first two mechanisms to relay information in a linear manner.

Now, of course, we know that signaling is far more complex, with different pathways operating via different molecular components and biochemical mechanisms. Modeling and experimentation have shown how such complex signaling mechanisms can carry out sophisticated signal processing. For example, protein kinase cascades can convert graded signals to ultrasensitive ones (Huang and Ferrell, 1996), feedbacks in cAMP signaling can produce temporal oscillations and the propagation of aggregation waves through cell populations (Loomis, 2014), and multi-protein receptor complexes can amplify weak signals to improve chemotaxis (Bray and Duke, 2004).

This enthusiasm to discover how signaling pathways performed signal processing had the effect of helping to obscure decades of pre-existing work which showed that many signaling systems act as linear transmitters (e.g. Knauer et al., 1984). However, quantitative biologists are finally on the case and now, in eLife, Harry Nunns and Lea Goentoro of Caltech report how three quite different metazoan signaling pathways – the canonical Wnt pathway, the EGF-ERK pathway, and the TGF-β pathway – act as linear transmitters (Nunns and Goentoro, 2018).

These pathways have disparate topologies and molecular mechanisms. Nunns and Goentoro found that established mathematical models of the three pathways can be simplified to demonstrate a linear relationship between the input and output. They also performed experiments to confirm this linearity for the Wnt and EGF-ERK pathways. Finally, they showed experimentally that perturbations predicted to break linearity (by inhibiting a protein kinase needed to destroy a protein called β-catenin in the Wnt pathway, or by blocking a key feedback in the EGF-ERK pathway) did exactly that.

The idea that ligand-bound receptors directly produced the cellular response endured for decades. However, once the complexity of key signaling pathways was realized, it became apparent that maintaining linearity in multistep pathways constituted a significant biochemical problem. Even simple binding reactions have nonlinear responses, and cascades of them result in increasingly sensitive responses (Strickland and Loeb, 1981). So far, researchers have identified three mechanisms that can prevent this distortion, thus ensuring a linear relationship between the cellular response and the percentage of ligand-bound receptors.

The first mechanism corrects an ultrasensitive response with negative feedback (Figure 1Bi). This control mechanism is well understood theoretically and is used in electronics to make voltage following amplifiers. Its biochemical implementations have also been explored theoretically and used to construct a genetic network with a linear relationship between input and output (Andrews et al., 2016; Nevozhay et al., 2009). Nunns and Goentoro's work is consistent with the idea that a kinase cascade in the EGF-ERK pathway acts as an ultrasensitive amplifier, and that feedback from ERK to the upstream kinase Raf corrects this distortion to linearize the downstream output.

The second mechanism involves the modulation of an unsaturated or unsaturable cycle (Figure 1Bii). Signaling via proteins that cycle between active and inactive forms is typically nonlinear because high input values deplete the supply of inactive proteins, which saturates the response. However, these systems exhibit linear signaling if the input values are kept low enough to have a minimal effect on the supply of inactive protein (Andrews et al., 2016): this appears to explain the linearity that Nunns and Goentoro found for TGF-β signaling, where a Smad protein cycles between active and inactive forms. Signaling systems also exhibit linear signaling if the cycle cannot be saturated, as is the case where the 'cycle' is one of synthesis and degradation followed by resynthesis; here, new proteins are synthesized from the intracellular pool of amino acids, which is effectively impossible to deplete. The linear signaling in the Wnt pathway observed by Nunns and Goentoro, in which β-catenin is rapidly created and destroyed, is consistent with this mechanism.

The third mechanism is a push-pull mechanism in which the active form of a signaling species stimulates downstream activity, while the nominally inactive form reduces downstream activity (Figure 1Biii; Andrews et al., 2016). Modeling and direct experiments have shown that this mechanism is responsible for the linear relationship between receptor occupancy and G protein activation in the yeast pheromone signaling system (Bush et al., 2016).

This work prompts two immediate thoughts. The first is to wonder why so many quantitative biologists neglected to study linear input-output signaling for so long. By the 1970s, pharmacologists routinely regarded the equivalence of percent ligand occupancy and percent of maximum downstream response as a criterion that a given molecule was likely to be the receptor for that ligand (Cuatrecasas, 1974). By the 1980s it was known that a number of other signaling systems, including the EGF-ERK system studied here (Knauer et al., 1984), had a linear relationship between input and output. Later, the linear relationship in the yeast pheromone signaling system was discovered: the consequences of this linearity included maximal information transmission and an increased robustness of the output to random downstream molecular events (Yu et al., 2008).

The second thought is to admire the established models of the three pathways studied by Nunns and Goentoro. These models were developed by different research groups, none of which set out to test linear signal transmission. The fact that the models nevertheless exhibit linearity, a behavior they were not built to reproduce, suggests that they represent the key molecules and interactions correctly.

This work also suggests topics for future research. The finding that these very different signaling pathways produce signaling without distortion is consistent with the idea that the consequences of such mechanisms were strongly selected for before the emergence of multicellular life. It would be interesting to build engineered systems that enable the careful study of the selective advantages that linear signaling mechanisms confer. Such systems might build on work which showed the linearity of downstream responses controlled by engineered autoregulating repressors (Nevozhay et al., 2009) that replicate the architecture of self-repressing bacterial operon systems (Brent and Ptashne, 1980; Smith and Magasanik, 1971). Combined experimental and analytical exploration of input-output behavior and its consequences in prokaryotic systems also seems in order. Finally, this work suggests that coupling such repressor-based systems to different eukaryotic signaling systems may define a path for building artificial signaling pathways that faithfully transduce human inputs into cellular outputs.
